# Mindfulness is associated with intrinsic functional connectivity between default mode and salience networks

**DOI:** 10.3389/fnhum.2015.00461

**Published:** 2015-08-25

**Authors:** Anselm Doll, Britta K. Hölzel, Christine C. Boucard, Afra M. Wohlschläger, Christian Sorg

**Affiliations:** ^1^Department of Neuroradiology, Technische Universität München TUMMunich, Germany; ^2^Department of Psychiatry, Technische Universität München TUMMunich, Germany; ^3^TUM-Neuro Imaging Center of Klinikum Rechts der Isar, Technische Universität München TUMMunich, Germany; ^4^Graduate School of Systemic Neurosciences, Ludwig-Maximilians-UniversityMunich, Germany; ^5^Department of Neurology, Technische Universität München TUMMunich, Germany; ^6^Department of Nuclear Medicine, Technische Universität München TUMMunich, Germany

**Keywords:** resting state, connectivity, mindfulness, fMRI, salience network, central executive network, default network

## Abstract

Mindfulness is attention to present moment experience without judgment. Mindfulness practice is associated with brain activity in areas overlapping with the default mode, salience, and central executive networks (DMN, SN, CEN). We hypothesized that intrinsic functional connectivity (iFC; i.e., synchronized ongoing activity) across these networks is associated with mindfulness scores. After 2 weeks of daily 20 min attention to breath training, healthy participants were assessed by mindfulness questionnaires and resting-state functional MRI. Independent component analysis (ICA) of imaging data revealed networks of interest, whose activity time series defined inter-network intrinsic functional connectivity (inter-iFC) by temporal correlation. Inter-iFC between subnetworks of the DMN and SN—and inter-iFC between subnetworks of the SN and left CEN at trend—was correlated with mindfulness scores. Additional control analyses about visual networks’ inter-iFC support the specificity of our findings. Results provide evidence that mindfulness is associated with iFC between DMN and SN. Data suggest that ongoing interactions among central intrinsic brain networks link with the ability to attend to current experience without judgment.

## Introduction

Mindfulness refers to attending to present moment experience without judging occurring feelings or thoughts (Bishop et al., 2004). Mindfulness practice such as meditation has proved beneficial for well-being (Carmody, 2009), and is an efficient element of treatments for mental disorders (Hofmann et al., 2010). Mindfulness practice typically recruits a number of brain regions, mainly prefrontal, parietal and subcortical brain areas (Creswell et al., 2007; Frewen et al., 2010; Hasenkamp et al., 2012; Dickenson et al., 2013). For example, Dickenson et al. (2013) found activations in dorso-medial prefrontal cortex (dmPFC), anterior cingulate cortex (ACC), insula, and temporo-parietal junction during a controlled focused breathing task—the most prominent first technique in teaching mindfulness to novices. Due to its widespread activation pattern, distributed functional brain networks have been suggested as critical neural correlates of mindfulness practice. For example, Hasenkamp and Barsalou (2012) recently identified four different mental states during meditation with each state being preferentially related to activity in different intrinsic brain networks: focus on the present experience was most strongly related to dorso-lateral prefrontal cortex activation of the central executive network (CEN), mind wandering was associated with the default mode network (DMN), awareness of mind wandering was linked with activation in the salience network (SN), and a shift of attention back towards focus on the present experience was again linked with the right dorsolateral prefrontal cortex (DLPFC) and right posterior parietal cortex with both regions being part of the CEN.

Intrinsic brain networks such as the DMN, SN, and CEN are characterized by coherent ongoing activity at infra-slow frequencies and are often studied during resting state. These networks are believed to subserve specific cognitive functions like attentional control or core affect (Fox and Raichle, 2007) as their patterns of coherent ongoing activity overlap and reflect the activation patterns observed during goal directed behavior (Lewis et al., 2009; Smith et al., 2009; Berkes et al., 2011; Riedl et al., 2011). Multiple experiments have shown that correlated resting-state activity (i.e., intrinsic functional connectivity—iFC) within the DMN, SN, CEN, respectively, is associated with mindfulness (Kilpatrick et al., 2011; Hasenkamp and Barsalou, 2012; Shaurya Prakash et al., 2013; Taylor et al., 2013; Berkovich-Ohana et al., 2014). However, previous studies focused mainly on within-network functional connectivity, ignoring that additional networks are involved in mindfulness and particularly that these networks interact with each other (see Kilpatrick et al., 2011; Froeliger et al., 2012). Based on Hasenkamp and Barsalou’s (2012) model, we hypothesized that the ability of mindfulness is coded in ongoing inter-network interactions between DMN, SN, and CEN. To test this hypothesis we investigated the inter-network functional connectivity during resting state (inter-iFC) of the SN, DMN, and CEN in 26 healthy controls that were trained in breath awareness for 2 weeks prior to scanning, and correlated these values with participants’ mindfulness scores of psychometric assessment.

## Materials and Methods

### Participants

Twenty six right-handed meditation naïve volunteers that were free from past and present neurological and psychiatric disorders participated in the study (10 males, mean age (±SD) = 26.9 ± 4.6). The study was approved by the ethical committee of the Technische Universität München. All participants provided written informed consent prior to the experiment and received a monetary compensation of expenses for participation.

### Mindfulness Ability

To ensure mindfulness ability, participants received 20 min of audio-guided training in attention to breath, based on a meditation as taught in a mindfulness based stress reduction (MBSR) program (Hölzel, 2012) daily for 2 weeks. Mindfulness ability was measured with the Mindful Attention and Awareness Scale (MAAS; Brown and Ryan, 2003) and the Freiburg Mindfulness Inventory (FMI; Walach et al., 2006). While the MAAS focuses on the presence or absence of awareness of what is occurring in the present, the FMI assesses the accepting and curious attitude towards this experience that is inherent to mindfulness. Both questionnaires have been shown to hold good internal consistency (Cronbach’s *α* = 0.82 and 0.86, respectively) and validity.

### Functional MRI: Data Acquisition and Analysis

For imaging, participants were instructed to remain still with eyes closed and to not fall asleep during acquisition. All participants reported to not have fallen asleep during the scanning session.

### Imaging Acquisition

Magnetic resonance imaging was performed on a 3-T whole body MR scanner (Verio, Siemens, Germany) using a standard head coil. For co-registration of functional data, T1-weighted anatomical data were obtained from each subject by using a magnetization-prepared rapid acquisition gradient echo sequence [MP-RAGE, time to echo (TE) = 4 ms, repetition time (TR) = 9 ms, time for inversion (TI) = 100 ms, flip angle = 5°, field of view (FoV) = 240 mm × 240 mm, matrix = 240 × 240, 170 slices, voxel size = 1 mm × 1 mm × 1 mm]. Functional data were collected using a contrast-gradient echo planar imaging (EPI) sequence (TE = 35 ms, TR = 2000 ms, flip angle = 90°, 35 slices, slice thickness = 3 mm, and 0 mm interslice gap).

### fMRI Data Analysis

Preprocessing and analysis of imaging data was carried out with SPM8 (Wellcome Department of Cognitive Neurology, London, UK). After coregistration and segmentation, T1-weighted structural images were normalized to a standard T1 template in MNI space with a 1 × 1 × 1 mm resolution. After discarding the first three volumes, preprocessing of T2*-weighted functional images included slice timing, spatial realignment to the first image of the run, normalization to SPM8’s EPI template in the Montreal Neurological Institute (MNI) space, resampling to 3 × 3 × 3 mm and smoothing with an 8 mm full width at half maximum (FWHM) Gaussian filter.

To define intrinsic networks, we applied high-model-order independent component analysis (ICA) to the preprocessed data by using the GIFT-toolbox^1^ with the infomax algorithm implemented in Matlab (Calhoun et al., 2001). Data were decomposed into 75 spatial independent components (IC), correspondent with a framework for high-model-order decomposition (Abou Elseoud et al., 2011; Allen et al., 2011). High-model-order ICA approaches of about 70 components yield IC, which are in optimal accordance with known anatomical and functional segmentations (Damoiseaux et al., 2006; Kiviniemi et al., 2009; Smith et al., 2009). Data were concatenated and reduced by two-step principal component analysis (PCA), followed by independent component estimation with the infomax-algorithm. We subsequently ran 40 ICAs (ICASSO) to ensure stability of the estimated components (Himberg et al., 2004). This results in a set of average group components which are then back reconstructed into single subject space, each represented by a spatial map of z-scores reflecting the within-network iFC and one associated time course of BOLD-signal fluctuations representative for this IC.

To select the IC reflecting networks of interest in an automated and objective way, we conducted multiple spatial regressions of 75 IC’ spatial maps on T-maps of intrinsic connectivity networks (ICNs) as described in Allen et al. (2011). These T-maps were generated by the identical ICA approach as performed in the current study based on 603 healthy adolescents and adults and were made available online by the Medical Image Analysis Lab (MIALAB).^2^ For each ICN, the independent component with the largest correlation coefficient was chosen. According to our hypothesis, we restricted our selection to ICNs, which were characterized as part of either SN, DMN, or CEN (ICs 25, 34, 50, 53, 55, 60, 68 in Allen et al., 2011), resulting in a total of seven ICNs for further analysis.

To define outcome measures of inter-network iFC, we performed Pearson correlation analyses for all of these networks resulting in 21 correlations per participant. Pearson correlation coefficients were transformed into *z*-values via Fisher r-to-z transformation. Subsequently, we correlated these z-values with the two measures of mindfulness ability across participants, respectively, and evaluated statistical significance of results via *t*-tests (*p* < 0.05, Bonferroni corrected for 21 tests with corrected *p*-value *p* < 0.0024).

In order to test the specificity of the link between inter-iFC and mindfulness for neuro-cognitive key networks, we additionally selected three visual occipital networks from Allen et al. (2011): IC 46, 64 and 67 and performed the identical analysis, including computation of inter-iFC between these visual networks and associations with mindfulness scores. We chose occipital visual networks, since a previous study demonstrated intra-network connectivity changes in such a visual network after 8 week mindfulness training (Kilpatrick et al., 2011), suggesting that its inter-network connectivity might be linked with trait mindfulness, too. Furthermore, we performed a similar analysis for the inter-iFC between the visual and neuro-cognitive networks, respectively. That is we computed inter-iFC and its association with mindfulness scores.

## Results

### Psychometric Assessment of Mindfulness

Mindfulness was assessed using the MAAS and the FMI. Mean scores were 60.9 (SD = 8.5) for the MAAS and 37.5 (SD = 3.1) for the FMI. The FMI contained two outliers (based on the outlier labeling rule; Hoaglin and Iglewicz, 1987) that were excluded from all analyses pertaining to FMI scores. The correlation between the questionnaires was significant (*r* = 0.45, *p* < 0.02).

### Intrinsic Networks and Inter-Network Connectivity

**Figure 1:**
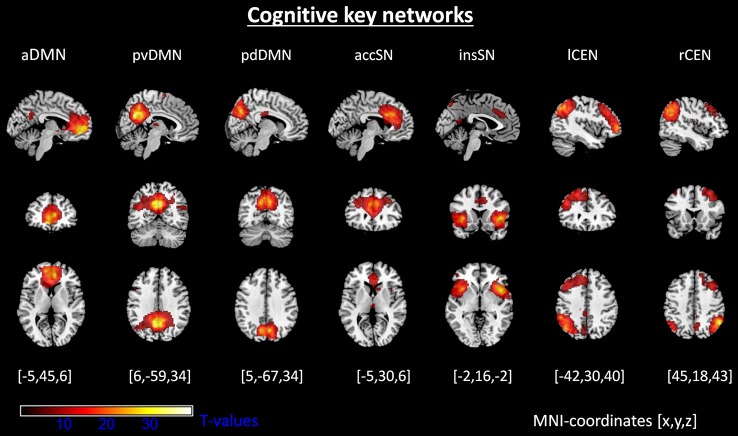
**Intrinsic networks of interest**. Shown are spatial maps of one-sample *t*-tests (voxel wise family wise error corrected, *p* < 0.05). Abbreviations: aDMN, anterior default mode network; pvDMN, posterior ventral default mode network; pdDMN, posterior dorsal default mode network; accSN, cingular salience network; insSN, insula salience network; lCEN, left central executive network; rCEN, right central executive network.

**Figure 2:**
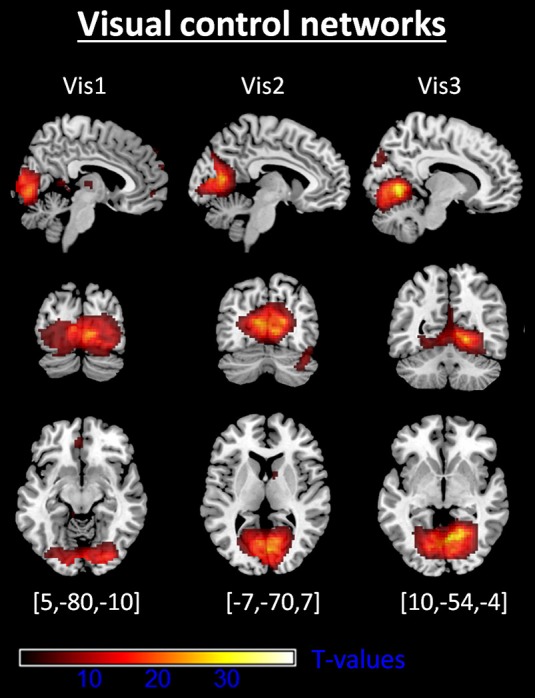
**Intrinsic visual control networks**. Shown are spatial maps of one-sample *t*-tests (voxel wise family wise error corrected, *p* < 0.05). Abbreviations: Vis1, visual network 1; Vis2, visual network 2; Vis3, visual network 3. coordinates are given with respect to Montreal Neurological Institute standard space.

Automated component selection revealed seven components of interest for each individual. Selected components matched previous results of SN, DMN, and CEN (Allen et al., 2011; Figure 1; Table 1; *p* < 0.05, FWE-corrected). The SN was represented in two components, comprising ACC (accSN) and bilateral anterior insula (insSN). DMN was represented in three components [anterior DMN (aDMN; medial prefrontal cortex), posterior-ventral DMN (pvDMN; posterior cingulate cortex, PCC) and posterior-dorsal DMN (pdDMN; precuneus)]. CEN was represented in two components, comprising two lateralized fronto-parietal networks corresponding to left (lCEN; left middle and superior frontal cortex, and inferior parietal lobule), and right CEN (rCEN; right middle and superior frontal gyri, right angular gyrus, and right inferior parietal lobule). The three visual control networks were located in medial and lateral occipital cortex and matched well with the templates in Allen et al. (2011; Figure 2).

**Table 1:** **Intrinsic brain networks of interest**.

Network	Region	Voxel: *t*-value	*P*-value	Cluster size	Peak MNI-coordinates		
					*x*	*y*	*z*
aDMN	Left anterior cingulate cortex	30.7	<0.01	2047	−6	53	1
	Left middle orbital gyrus	30.6	<0.01		−6	47	−8
	Right anterior cingulate cortex	29	<0.01		6	47	7
	Left posterior cingulate cortex	11.6	<0.01	87	0	−52	28
pvDMN	Right precuneus	39.98	<0.01	2632	6	61	28
	Left posterior cingulate cortex	28.34	<0.01		−6	−52	28
	Left precuneus	34.4	<0.01		−6	−61	34
	Right middle cingulate cortex	28.7	<0.01		12	−49	34
pdDMN	Left precuneus	27.3	<0.01	1275	−12	−67	34
	Right precuneus	26.3	<0.01		9	−70	37
	Left cuneus	24.8	<0.01		−6	−76	34
accSN	Right middle cingulate cortex	27.1	<0.01	2714	9	26	31
	Left anterior cingulate cortex	26.7	<0.01		−6	14	28
insSN	Right insular lobe	34.2	<0.01	891	45	14	1
	Left insular lobe	25.8	<0.01	813	−36	17	1
	Left inferior frontal gyrus	18.8	<0.01		−39	14	−5
	Right middle cingulate cortex	11.5	<0.01	280	9	11	40
	Left middle cingulate cortex	9.7	<0.01		0	26	34
lCEN	Left middle frontal gyrus	26.2	<0.01	1808	−36	56	7
	Left superior frontal gyrus	19.9	<0.01		−24	13	63
	Left inferior parietal lobule	18.9	<0.01		−54	−49	43
	Left angular gyrus	26.9	<0.01	1342	−42	−58	46
	Left inferior temporal gyrus	19.5	<0.01	104	−54	−49	−8
	Left middle cingulate cortex	10.0	<0.01		−3	−34	43
rCEN	Right inferior parietal lobule	34	<0.01	1027	54	−58	40
	Right angular gyrus	32.9	<0.01		51	−58	31
	Right middle frontal gyrus	16.8	<0.01	603	33	17	52
	Right superior frontal gyrus	13.5	<0.01		21	26	55
	Left angular gyrus	17.2	<0.01	338	−42	−58	34
	Left inferior parietal lobule	14.5	<0.01		−48	−55	46
	Right precuneus	13.5	<0.01	216	6	−58	37
	Right middle cingulate cortex	13.5	<0.01		6	−46	34

*Shown are results of one-sample t-tests of resting state brain intrinsic brain networks after independent component analysis with 75 components (voxel wise family wise error correction for multiple comparisons, p < 0.05). Abbreviations: DMN, default mode network; SN, salience network; CEN, central executive network; aDMN, anterior default mode network; pvDMN, posterior ventral default mode network; pdDMN, posterior dorsal default mode network; accSN, cingular salience network; insSN, insula salience network; lCEN, left central executive network; rCEN, right central executive network*.

From the seven intrinsic networks of interest and the three control networks, we extracted the network time courses and defined inter-iFC via Pearson’s correlation (see Table 2 for mean correlation coefficients of networks of interest and Table 4 for visual control networks).

**Table 2:** **Functional connectivity between intrinsic networks**.

Network	insSN	pdDMN	pvDMN	aDMN	accSN	rCEN	lCEN
**insSN**	1.00	0.08	−0.14	0.08	0.14	−0.09	−0.05
**pdDMN**	0.08	1.00	0.29	0.25	0.06	0.23	0.24
**pvDMN**	−0.14	0.29	1.00	0.33	−0.17	0.37	0.24
**aDMN**	0.08	0.25	0.33	1.00	0.17	0.29	0.15
**accSN**	0.14	0.06	−0.17	0.17	1.00	0.08	0.01
**rCEN**	−0.09	0.23	0.37	0.29	0.08	1.00	0.25
**lCEN**	−0.05	0.24	0.24	0.15	0.01	0.25	1.00

*Shown are mean Pearson-correlation coefficients for each pair of networks of interest. Abbreviations: aDMN, anterior default mode network; pvDMN, posterior ventral default mode network; pdDMN, posterior dorsal default mode network; accSN, cingular salience network; insSN, insula salience network; lCEN, left central executive network; rCEN, right central executive network*.

### Association between Inter-iFC and Mindfulness Scores

**Figure 3:**
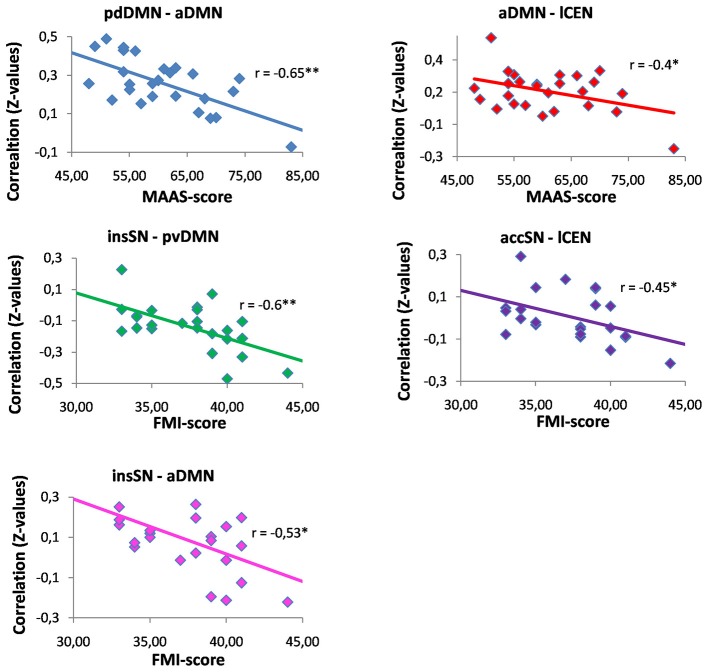
**Correlations between mindfulness ability and inter-network functional connectivity**. Abbreviations: MAAS, Mindful Attention and Awareness Scale; FMI, Freiburg Mindfulness Inventory; aDMN, anterior default mode network; pvDMN, posterior ventral default mode network; pdDMN, posterior dorsal default mode network; accSN, cingular salience network; insSN, insula salience network; lCEN, left central executive network; rCEN, right central executive network. *p < 0.05; **significant with Bonferroni correction for multiple comparisons.

**Figure 4:**
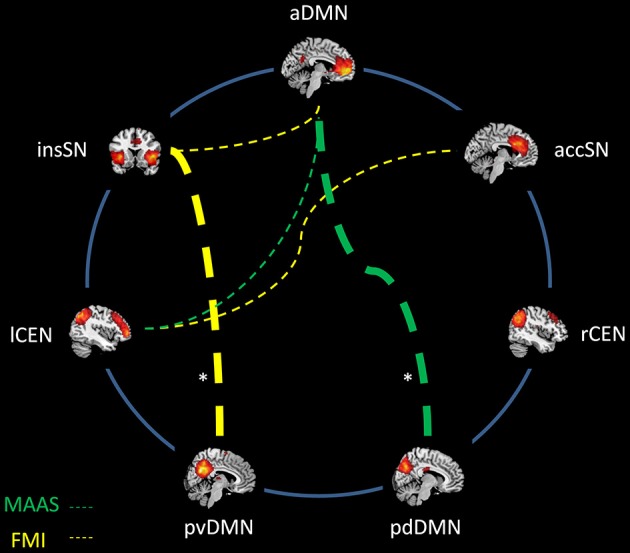
**Association between mindfulness ability and inter-network intrinsic functional connectivity (iFC)**. Networks are represented by spatial maps of one-sample *t*-tests (voxel wise family wise error corrected, *p* < 0.05). Dashed lines indicate negative correlations between mindfulness and connectivity strength. Abbreviations: MAAS, Mindful Attention and Awareness Scale; FMI, Freiburg Mindfulness Inventory; aDMN, anterior default mode network; pvDMN, posterior ventral default mode network; pdDMN, posterior dorsal default mode network; accSN, cingular salience network; insSN, insula salience network; lCEN, left central executive network; rCEN, right central executive network. *significant with Bonferroni correction for multiple comparisons.

After Fisher r-to-z transformation, we correlated z-scores of inter-iFC with the mindfulness scores of both FMI and MAAS (Pearson correlation, *p* < 0.05, Bonferroni corrected for 21 comparisons, corrected threshold *p* < 0.0024; Figures 3, 4; Table 3). We found significant negative correlations between FMI and inter-iFC of insSN and pvDMN (*r* = −0.60, *p* < 0.002). In addition, inter-iFC between the aDMN and the pdDMN was significantly negatively correlated with the MAAS scores (*r* = −0.65, *p* < 0.001). At border significance (i.e., *p* < 0.05, but not surviving correction for multiple testing), we found negative associations between MAAS and inter-iFC of aDMN and lCEN (*r* = −0.40, *p* < 0.045), FMI and inter-iFC of insSN and aDMN (*r* = −0.53, significance *p* < 0.008), as well as FMI and inter-iFC between accSN and lCEN (*r* = −0.45, *p* < 0.03).

**Table 3:** **Pearson’s correlation coefficients between inter-network intrinsic functional connectivity (inter-iFC) and mindfulness ability**.

	rCEN/lCEN	aDMN/lCEN	aDMN/rCEN	aDMN/accSN	pvDMN/lCEN
*r*-value FMI	0.05	0.29	−0.06	0.02	0.13
*P*-value	0.82	0.16	0.79	0.94	0.54
*r*-value MAAS	−0.12	−0.40	−0.18	0.02	−0.33
*P*-value	0.55	0.05*	0.39	0.92	0.10
	**pdDMN/lCEN**	**pdDMN/rCEN**	**pdDMN/aDMN**	**pdDMN/pvDMN**	**pdDMN/accSN**
*r*-value FMI	0.13	0.01	−0.36	−0.16	0.14
*P*-value	0.53	0.98	0.09	0.45	0.50
*r*-value MAAS	0.24	−0.13	−0.65	−0.10	0.03
*P*-value	0.24	0.51	<0.01**	0.63	0.87
	**pvDMN/rCEN**	**pvDMN/aDMN**	**pvDMN/accSN**	**accSN/lCEN**	**accSN/rCEN**
*r*-value FMI	−0.05	−0.09	0.11	−0.45	−0.14
*P*-value	0.80	0.69	0.61	0.03*	0.53
*r*-value MAAS	−0.09	−0.30	0.04	0.06	−0.11
*P*-value	0.66	0.14	0.83	0.79	0.59
	**insSN/lCEN**	**insSN/rCEN**	**insSN/aDMN**	**insSN/pvDMN**	**insSN/accSN**
*r*-value FMI	−0.04	−0.34	−0.53	−0.60	−0.02
*P*-value	0.86	0.10	0.01*	<0.01**	0.94
*r*-value MAAS	0.33	0.08	−0.13	−0.14	0.02
*P*-value	0.09	0.69	0.53	0.49	0.93
	**insSN/pdDMN**
*r*-value FMI	−0.09
*P*-value	0.67
*r*-value MAAS	0.30
*P*-value	0.14

Abbreviations: MAAS, Mindful Attention and Awareness Scale; FMI, Freiburg Mindfulness Inventory; aDMN, anterior default mode network; pvDMN, posterior ventral default mode network; pdDMN, posterior dorsal default mode network; accSN, cingular salience network; insSN, insula salience network; lCEN, left central executive network; rCEN, right central executive network. *p < 0.05; **significant with Bonferroni correction for multiple comparisons for 21 multiple comparisons (corrected threshold p = 0.0024).

To evaluate the influence of sex on the association between mindfulness and inter-network connectivity, we re-analyzed the dependence between inter-iFC and MAAS and FMI, respectively, by the use of partial correlation analysis with sex as additional control variable. In partial correlation analysis, the dependence between two variables (i.e., inter-iFC and mindfulness score) is evaluated while controlling for the influence of further variables (i.e., sex) on both variables of interest, respectively. While almost all results concerning the link between inter-iFC and mindfulness scores changed only marginally, merely the association between MAAS and inter-iFC of aDMN and lCEN, which was at-trend (*p* = 0.05) in the analysis without control for sex, lost its trend to significance. This finding indicates that the observed link between mindfulness and inter-iFC between SN and DMN is largely independent from sex differences.

For the association between mindfulness scores and inter-iFC among visual control networks, we found no significant correlation (Table 4). The analysis of the association between mindfulness scores and inter-iFC between visual and neuro-cognitive networks of interest yielded one at trend result (Table 5). The correlation between FMI and the connectivity between the anterior DMN and a secondary visual network (reflecting IC 67 component of Allen et al. (2011) and covering mainly the lingual gyrus) had a Pearson’s coefficient of *r* = −0.53 with a *p*-value of 0.01. This *p*-value did not survive correction for multiple testing via Bonferroni for corrected threshold of *p* < 0.0024.

**Table 4:** **Control analysis: Inter-network connectivity and associations with mindfulness of visual networks**.

Network	Vis1/Vis2	Vis1/Vis3	Vis2/Vis3
Inter-iFC	0.27	0.43	0.38
Correlation with FMI	−0.34	−0.33	−0.38
*p*-value	0.1	0.11	0.07
Correlation with MAAS	−0.18	−0.12	−0.09
*p*-value	0.38	0.57	0.67

*Shown are mean Pearson-correlation coefficients for each pair of visual control networks and below corresponding correlations of inter-iFC and mindfulness scores. Abbreviations: Vis1, visual network 1; Vis2, visual network 2; Vis3, visual network 3; FMI, Freiburg Mindfulness Inventory; MAAS, Mindful Attention and Awareness Scale*.

**Table 5:** **Control analysis: inter-network connectivity between visual networks and neuro-cognitive key networks and associations with mindfulness ability**.

Network	insSN	pdDMN	pvDMN	aDMN	accSN	lCEN	rCEN
**Visual 1**	0.04	0.30	0.17	0.13	−0.08	0.15	−0.05
Correlation with FMI	−0.07	−0.12	0.02	−0.36	0.35	0.16	0.31
*p*-value	0.75	0.57	0.93	0.08	0.09	0.45	0.14
Correlation with MAAS	0.21	0.00	−0.22	−0.09	0.28	−0.13	0.10
*p*-value	0.31	0.99	0.27	0.66	0.16	0.51	0.64
**Visual 2**	0.05	0.29	0.23	0.09	−0.18	0.11	>−0.01
Correlation with FMI	−0.24	−0.11	−0.27	0.02	0.04	0.10	0.20
*p*-value	0.25	0.61	0.20	0.94	0.84	0.64	0.36
Correlation with MAAS	0.14	0.00	−0.23	−0.27	−0.22	0.35	−0.18
*p*-value	0.51	0.99	0.26	0.17	0.28	0.08	0.37
**Visual 3**	0.10	0.23	0.12	<0.01	−0.21	0.04	−0.07
Correlation with FMI	−0.17	−0.09	−0.14	−0.53	0.28	0.28	0.06
*p*-value	0.43	0.69	0.52	0.01*	0.18	0.18	0.79
Correlation with MAAS	0.13	0.08	−0.18	−0.16	0.09	0.15	<0.01
*p*-value	0.53	0.69	0.37	0.43	0.65	0.47	0.98

Shown are mean Pearson-correlation coefficients for each pair of networks of interest and three control networks. In addition, correlations and p-values of the respective inter network connectivity and mindfulness scores are shown. Abbreviations: aDMN, anterior default mode network; pvDMN, posterior ventral default mode network; pdDMN, posterior dorsal default mode network; accSN, cingular salience network; insSN, insula salience network; lCEN, left central executive network; rCEN, right central executive network. Visual 1, visual network; Visual 2, visual network 2; Visual 3, visual network 3; *p-value significant without bonferroni correction (cutoff p = 0.0024).

## Discussion

The present study investigated the association between mindfulness and functional connectivity of intrinsic brain activity among three central neurocognitive networks (inter-iFC): the SN, DMN and CEN. Inter-iFC between DMN and SN—and inter-iFC between SN and CEN at trend—was correlated with mindfulness scores. Results suggest that mindfulness is significantly associated with ongoing network interactions of central neurocognitive networks.

### Relationship between Mindfulness and Inter-Network Connectivity

We found negative correlations of mindfulness ability and inter-iFC among SN, DMN and CEN (Figures 3, 4; Table 3). These correlations were significant for the negative inter-iFC between insSN and pvDMN. This result was specific for neuro-cognitive key networks, since control analysis for visual networks inter-network connectivity did not yield significant results (Table 5). Furthermore it was independent from sex differences, for which we controlled in additional control analyses. The increase in anti-correlation between the insSN and pvDMN for more mindful individuals replicates previous findings (Kilpatrick et al., 2011). Kilpatrick et al. (2011) compared intrinsic brain networks in participants of an 8 week course of mindfulness stress reduction (MBSR) to waitlist controls during a mindful resting state. After training in MBSR, participants showed increased anti-correlation between a region within the cuneus (part of the pvDMN) and their equivalent of the SN (Kilpatrick et al., 2011). In another study, Hasenkamp and Barsalou (2012) performed a whole brain resting state FC analysis of a network comprising bilateral posterior cingulate cortex (PCC) and ventro-medial prefrontal cortex (vmPFC) regions of interest, representing the DMN, and compared this between high and low practice meditators. Supporting the present study, they found a decrease in correlation between the hubs of the DMN and the insSN, in high practiced meditators (Hasenkamp and Barsalou, 2012). The SN has been hypothesized to be involved in the detection and evaluation of motivationally salient stimuli, i.e., stimuli with relevance for the organism (Seeley et al., 2007) and in controlling interactions between the DMN and CEN (Sridharan et al., 2008), while the DMN is involved in memory, self-related, and social processing (for a review, see Buckner et al., 2008). Furthermore, the DMN has been shown to be activated during mind wandering (Mason et al., 2007; Hasenkamp et al., 2012). Anti-correlation could be interpreted as a clearer distinction between the networks which might result in better effective connectivity (Clare Kelly et al., 2008; Deco et al., 2009; Lewis et al., 2009). Following this line of thought, an increased anti-correlation between insSN and the pvDMN might indicate improved sensitivity to mind wandering in more mindful individuals. This anti-correlation-based connectivity between the insSN and pvDMN was particularly related to the FMI, which is focused on measuring the accepting stance towards all experience that is inherent to mindfulness (Walach et al., 2006). The pvDMN included the PCC, which is active during personal evaluations (Whitfield-Gabrieli et al., 2011) and emotional processing (Kober et al., 2008), which has been hypothesized to be related to the meditative experience (Brewer and Garrison, 2014). Together with the insSN’s involvement in directing attention to salient stimuli (Seeley et al., 2007), stronger negative correlation between pvDMN and insSN might indicate a less evaluative stance during rest towards experience, which would fit well with the topic of the FMI measuring acceptance (Walach et al., 2006).

Interestingly, participants of the studies cited above (Kilpatrick et al., 2011; Hasenkamp and Barsalou, 2012) had considerable more experience with meditation than the participants of the present study, but show a comparable pattern of changes in connectivity. It seems thus, that changes in connectivity between the SN and the DMN are among the first to become apparent during mindfulness training that could be extended by changes in connectivity of e.g., task positive or attentional networks.

In addition to the above mentioned results, we found several results that were significant only at trend level. The connection of the aDMN and the lCEN being sensitive to mindfulness may confirm results found previously for experienced meditators (Hasenkamp and Barsalou, 2012; Taylor et al., 2013) although these studies found an increase in connectivity rather than a reduction for experienced meditators. The CEN is thought to be involved in the redirection of attention (Corbetta and Shulman, 2002), while the aDMN, specifically the ventro-medial PFC is involved in self-related processing (e.g., Andrews-Hanna et al., 2010). The present data might suggest that more mindful individuals may have a lower correlation between these networks, which could indicate an increased switching of attention away from self-related towards e.g., more sensory focused processing.

Moreover, we found a relationship of mindfulness scores and connectivity of the lCEN and the accSN, which had not been investigated previously. The accSN has been associated with processing of emotions, cognition and action inhibition (Smith et al., 2009). Speculating towards a reduced connectivity between the lCEN and accSN in more mindful individuals, it might be possible that this emphasizes conscious attentional processing over emotional value based evaluation of stimuli. Together, the present data show that connectivity in resting state networks may be sensitive for mindfulness effects. Concerning the direction of mindfulness effect (i.e., more or less inter-iFC), there are a number of inconsistencies about certain connections and the directionality in the literature and consequently when comparing our findings with those of previous studies. Possible explanations for these irregularities could be factors related to study design that can influence resting state connectivity. Activations during tasks performed immediately before a resting sate scan have been shown to carry over into resting state connectivity (Tambini et al., 2010). This might e.g., occur if meditation is performed or trained immediately before the resting state scan. Researchers should consider an appropriate break between task or training and rest scans. Similarly, we cannot control for what participants actually do during the resting state scan. Especially in meditation research this aspect is critical as meditation itself is performed under conditions very similar to resting and some individuals might by default engage in focused attention or other meditation during rest. This aspect is seldom discussed and should receive additional attention. Future studies should carefully instruct participants before performing a resting state scan making sure participants do not enter a meditation state.

### Connectivity Between the Anterior and Posterior DMN

The present study showed that the connectivity of the anterior and the posterior parts of the DMN was sensitive to mindfulness ability (Figure 3). The DMN has most strongly been in the focus of mindfulness research and a number of studies have reported connectivity changes related to mindfulness (Brewer et al., 2011; Jang et al., 2011; Kilpatrick et al., 2011; Hasenkamp and Barsalou, 2012; Shaurya Prakash et al., 2013; Taylor et al., 2013). The present study showed decreased connectivity in individuals with higher mindfulness ability between the aDMN and pdDMN. This result is in accordance with the results by Hasenkamp and Barsalou (2012) who found that a region in mPFC/ACC showed decreased connectivity with the PCC, the main hub of the pDMN, in mindfulness experts compared to novices. However, this result seems to contradict two previous results, which showed increased connectivity in more mindful individuals (Shaurya Prakash et al., 2013) and in experienced meditators (Jang et al., 2011). Yet, while one of these studies focused on elderly participants (Shaurya Prakash et al., 2013) the other used a region of interest approach to identify the DMN, which does not differentiate between posterior and anterior DMNs (Jang et al., 2011). These differences in study design might explain the difference in the results. In addition, other studies did not find any relationship for the connection of the anterior and posterior parts of the DMN with mindfulness (Kilpatrick et al., 2011; Taylor et al., 2013). The main hubs of the DMN are the precuneus/PCC (posterior DMN) and the vmPFC/ACC region (anterior DMN). The precuneus, is involved in the affective relevance of a given stimulus and is a critical structure for conscious processing (for a review, see Vogt and Laureys, 2005) while the vmPFC is involved in self-referential processing (Andrews-Hanna et al., 2010). A decreased connectivity of the aDMN and pDMN might indicate that more mindful individuals interpret the affective relevance of a given stimulus as less self-related. This is also supported by the association of the MAAS questionnaire with this connectivity. The MAAS focuses on measuring the ability to consciously perceive the present moment (Brown and Ryan, 2003). This present moment experience has been associated with a deactivation of the PCC/Precuneus (Garrison et al., 2013) area and with activations in dorso medial (Hölzel et al., 2007; Dickenson et al., 2013) and lateral prefrontal regions (Brefczynski-Lewis et al., 2007). Thus, this would speak for a reduced synchrony of the antDMN and the pDMN regions during this experience, which could transition into a stronger decoupling of these parts of the DMN network in more mindful individuals during rest. Instead, these regions may be coupled more strongly to either lateral parietal or DLPFC in expert meditators. e.g., Brewer et al. (2011) found increased connectivity between the PCC, dorsal ACC and DLPFC in participants with more meditation experience both during rest and during different kinds of meditation. The authors interpreted these results as an at baseline increased connectivity and activity of task positive control regions together with reduced activation of the DMN in experienced meditators regardless of condition. Other authors have argued for a coactivation of the aDMN together with inferior parietal regions during rest, which might reduce distractibility by mind wandering in experienced meditators (Hasenkamp and Barsalou, 2012). Our data are more in accordance with the model by Hasenkamp and Barsalou (2012), which suggests a critical interplay between medial DMN and lateral CEN for engaging attention on present experience. Instead of being engaged in mind-wandering which results in activation of the anterior and posterior DMN (Mason et al., 2007), regions in the dmPFC might be responsible for focusing attention back to present experience likely reflected by stronger anti-correlated coupling between CEN and DMN. Future studies will have to further clarify the directionality of connectivity between the anterior DMN, posterior DMN, and attention-relevant regions of frontal and parietal CEN and how this is related to meditation experience and mindfulness disposition.

### Limitations

To evaluate the key findings of our study, some limitations have to be considered. Firstly, our approach to study the relevance of inter-iFC in neuro-cognitive key networks for mindfulness is a correlation-based approach (i.e., we linked mindfulness scores with inter-iFC via linear correlation). Whether increased negative iFC between SN and DMN, which we demonstrated to correlate with mindfulness ability, is causal for the variability in mindfulness ability is not addressable by a correlation-based approach. To address such causal link between inter-iFC and mindfulness, controlled longitudinal training studies of mindfulness practice are necessary. In such studies, a controlled change in mindfulness ability can be linked with changes in inter-iFC of neuro-cognitive key networks. Secondly, given the wide range of functional domains, in which SN, CEN, and DMN are involved, it is hard to specify the behavioral implications of inter-iFC variability for mindfulness (i.e., which functional aspect of neuro-cognitive networks is relevant for mindfulness). Future studies combining resting-state and task fMRI with mindfulness might be helpful, if tasks are included that are part of mindfulness practice such as e.g., “focused attention to breathing.”

### Mindfulness as a Counterweight of Psychopathology

It is striking that mindfulness impacts the connectivity of three resting state networks (DMN, CEN, and SN) that have been shown to play a critical role in various psychopathologies (Menon, 2011). In detail, it has been hypothesized that the SN acts to control switching between DMN and CEN: depending on the demands of the task at hand the SN is hypothesized to balance activation in the other two networks (Sridharan et al., 2008; Menon and Uddin, 2010). Given that mindfulness has been shown to reduce psychopathological symptoms (Baer, 2003; Aldao et al., 2010; Hofmann et al., 2010), affecting the inter-iFC might be one possible pathway for beneficial effects of mindfulness in psychotherapy.

## Conclusion

Mindfulness is correlated with the inter-iFC of subnetworks of the DMN and SN. Specifically, more mindful individuals show a decreased correlation between the aDMN and pdDMN and stronger negative correlation between the insSN and pvDMN.

## Funding

This work was supported by the Elitenetzwerk Bayern (through the Ludwigs-Maximilians Universität and the Klinikum rechts der Isar, Technischen Universität München) for AD; the German Federal Ministry of Education and Research [BMBF 01EV0710 to A.M.W., BMBF 01ER0803 to CS], the Kommission für Klinische Forschung, Technische Universität München [KKF 8765162 to CS] and the Technische Universität München within the funding program Open Access Publishing.

## Conflict of Interest Statement

The authors declare that the research was conducted in the absence of any commercial or financial relationships that could be construed as a potential conflict of interest.

## Footnotes

^1^http://icatb.sourceforge.net

^2^http://mialab.mrn.org/data/hcp/RSN_HC_unthresholded_tmaps.nii
